# Effects of reduced winter duration on seed dormancy and germination in six populations of the alpine herb *Aciphyllya glacialis* (Apiaceae)

**DOI:** 10.1093/conphys/cou015

**Published:** 2014-05-30

**Authors:** G. L. Hoyle, H. Cordiner, R. B. Good, A. B. Nicotra

**Affiliations:** 1 Division of Evolution, Ecology and Genetics, Research School of Biology, Australian National University, Canberra, ACT 0200, Australia; 2 Australian National Botanical Gardens, Clunies Ross Street, Acton, Canberra, ACT 2601, Australia; 3 Fenner School of the Environment, Australian National University, Canberra, ACT 0200, Australia

**Keywords:** Alpine plants, Australia, climate change, global warming, morphological dormancy, physiological dormancy

## Abstract

The life stages of seed germination and seedling establishment play a vital role in maintaining plant populations and determining range dynamics of species. Thus, it is not surprising that specific germination requirements and dormancy mechanisms have evolved in all major angiosperm clades. In a rapidly changing climate, we face growing pressure to manage, conserve and restore native plant species and communities. To achieve these aims, we require solid knowledge of whether and how seed germination requirements and dormancy status vary between different populations of a given species and how germination strategies may be affected by warming climatic conditions. We assessed the effect of decreasing durations of cold stratification (i.e. conditions representing a shortened winter as predicted under climate change) on germination and dormancy of the alpine herb *Aciphylla glacialis*. Our results confirmed previous research showing that *A. glacialis* seeds possess physiological dormancy that can be alleviated by cold stratification. In addition, the results demonstrated that *A. glacialis* seeds have underdeveloped embryos at dispersal; these grow to germinable size following 4–9 weeks at both constant 5°C and 10–5°C (day–night) temperatures. We conclude that *A. glacialis* exhibits morphophysiological dormancy. Furthermore, we found that the final percentage germination and dormancy status varied significantly among natural populations and that this variation did not correlate with elevation at the site of seed origin. Seeds germinated following 6–8 weeks of cold stratification, and seedlings showed no detrimental effects as a result of shorter stratification periods. Together, these results suggest that reduced duration of winter is unlikely to have direct negative impacts on germination or early seedling growth in *A. glacialis.* The causes and implications of the population variation in germination traits are discussed.

## Abstract (precis)

Shortened winter durations, as expected under climate change, may affect seed germination and seedling growth of Alpine plant species, and these impacts may vary between populations, even within a single species.

## Introduction

The early stages of a plant's life cycle, namely seed germination and seedling establishment, play a vital role in maintaining plant populations (Fenner and Thompson, 2005) and determining range dynamics of species (Silvertown and Charlesworth, 2001; McGill *et al.*, 2006). The strong correlation between climate and recruitment has resulted in the evolution of specific germination requirements across many species (Baskin and Baskin, 2000; Fenner and Thompson, 2005), and dormancy mechanisms have evolved in all major angiosperm clades (Finch-Savage and Leubner-Metzger, 2006). Even within species, dormancy and germination requirements can vary (Cochrane *et al.*, in press). In a rapidly changing climate, we face growing pressure to manage, restore and conserve native plant species and communities both by predicting species responses *in situ* and by improving conservation outcomes using assisted migration and *ex situ* seed banking. To achieve these aims, we require solid knowledge of whether and how seed germination requirements and dormancy status vary between different populations of a given species and how the germination strategy of a species might be affected by climate change.

Species are not homogeneous across their natural distributions; species distributed across wide geographical or climatic ranges exhibit phenotypic variation in life history traits and physiological tolerances that reflect differences in selection pressures as well as random processes (Vergeer and Kunin, 2011). Studies indicate that variation in seed mass, germination rate and percentage, dormancy status, longevity and seedling growth rate occurs between populations and is sometimes correlated with environmental gradients (Cochrane *et al.*, in press). This trait variation within species can profoundly affect the response of species to environmental change (Suding *et al.*, 2008; Visser, 2008; Bolnick *et al.*, 2011), but the potential role of variation in germination strategies within species in determining the response of species to climate change and future distributions has received relatively little attention.

A species' germination strategy, or set of mechanisms regulating its seasonal emergence, arises from a combination of germination requirements and often dormancy mechanisms. Dormancy is an important seed characteristic and is characterized by the postponement of germination. For example, physiological dormancy prevents germination of freshly dispersed seeds despite adequate temperature and moisture conditions (Vleeshouwers *et al.*, 1995; Benech-Arnold *et al.*, 2000). The exact mechanism of physiological dormancy is not yet fully described (Nonogaki *et al.*, 2010), but it is generally understood to involve the effect of growth hormones upon embryonic extension. Abscisic acid and gibberellic acid maintain dormancy and promote germination, respectively, so that in a dormant seed, a feedback mechanism maintains a high abscisic acid-to-gibberellic acid ratio (Baskin and Baskin, 2004; Finch-Savage and Leubner-Metzger, 2006; Finkelstein *et al.*, 2008).

An underdeveloped embryo also prevents germination, and species exhibiting such embryos at the time of seed dispersal are referred to as having morphological dormancy (Martin, 1946; Nikolaeva, 1977). Morphologically dormant embryos require moist substrate and suitable temperatures in order to grow to a ‘critical’ size inside the seed before germination can commence. Germination in species exhibiting morphological dormancy is often controlled by an additional physiological dormancy mechanism, in which case the seed is described as having morphophysiological dormancy. Morphological dormancy is common among species of Apiacaeae, Ranunculaceae and Liliaceae species (Baskin and Baskin, 2000), including the Australian alpine herb *Psychrophila introloba* (Ranunculaceae; Wardlaw *et al.*, 1989), and low-temperature requirements vary among the different stages of dormancy alleviation in seeds with morphophysiological dormancy (Phartyal *et al.*, 2009).

Overall, dormancy mechanisms enable mature, viable seeds to avoid a germination response to temperature and rainfall events that are not conducive to subsequent seedling growth (Baskin and Baskin, 2004; Tielborger *et al.*, 2012). Thus, dormancy plays a key role in optimizing germination success by controlling the timing of germination (Leck *et al.*, 1989; Simons and Johnston, 2000; Donohue, 2005; Penfield and King, 2009).

Questions surrounding germination strategies and species variation therein are particularly important in habitats where potential for range shift is limited and where climate change projections are most extreme, such as alpine zones. High mountains are climatically stressful environments characterized by extreme weather events and low temperatures, short growing seasons (Billings and Mooney, 1968; Bliss, 1971) and annual fluctuations in seed production and quality (Chambers, 1995; Dullinger and Hulber, 2011). Such circumstances are likely to have favoured the evolution of mechanisms to ensure that alpine seed germination occurs not only in suitable microhabitats but also at optimal times of the year for onward seedling growth (Bell and Bliss, 1980; Körner, 2003).

Little is known about how changing climatic cues will alter alpine seed germination patterns, even though evidence from alpine areas around the world suggests that warming associated with climate change is occurring more rapidly above the treeline than at lower elevations, and that alpine areas are predicted to experience above-average warming in the future (Diaz and Bradley, 1997; Beniston, 2003; Hennessy *et al.*, 2003; Kullman, 2004; Green and Pickering, 2009). A consequence of this warming is shortened duration of stable, low temperatures and snow cover (Hughes, 2003; Green and Pickering, 2009). Given that temperature is a primary factor in stimulating germination and regulating changes in dormancy status (Vleeshouwers *et al.*, 1995; Benech-Arnold *et al.*, 2000), these climate changes may mean that germination strategies optimized for current alpine climes will be altered or disturbed in novel climates (Hanson and Weltzin, 2000; Hoyle *et al.*, 2008b; Klausmeyer and Shaw, 2009; Walck *et al.*, 2011). Even minor changes in the timing and placement of germination can have substantial implications for both individual species survival (Hovenden *et al.*, 2007) and community composition (Suttle *et al.*, 2007; Suggitt *et al.*, 2011). For some alpine species, climate warming could even lead to a shift from spring to autumn emergence, driven primarily by changes in seed dormancy status and resulting in major implications for species currently adapted to emergence in spring (Mondoni *et al.*, 2012). Such predictions make it likely that there will be mismatching in the timing and occurrence of germination for many species, similar to that apparent for other biotic interactions (Parmesan and Yohe, 2003; Walther, 2010).

In the present study, we examined mechanisms regulating the germination strategy of *Aciphylla glacialis* (F. Muell.) Benth. (Apiaceae), a dioecious perennial herb endemic to the Australian alpine region. *Aciphylla glacialis* seeds possess physiological dormancy that can be alleviated by cold stratification, and populations vary in final percentage germination (Venn and Morgan, 2009; G. L. Hoyle *et al.*, unpublished observations). Germination requirements and timing of seedling emergence have been studied in a large number of Apiaceae species of northern temperate climates, revealing that, for many species, germination is programmed to occur in late winter or spring, after dormancy alleviation during winter (Vandelook *et al.*, 2009 and references therein). Apiaceae species typi cally produce seeds containing underdeveloped embryos at dispersal (Martin, 1946), although this has not been reported for *A. glacialis*. Therefore, we asked the following questions. (i) Does seed dormancy status vary among natural populations? (ii) Is a shortened alpine winter likely to affect dormancy alleviation and thus, germination success? (iii) Do seeds that receive reduced cold periods for dormancy alleviation show any effect on subsequent seedling growth? (iv) Are *A. glacialis* seed embryos underdeveloped at dispersal and, if so, how quickly and in what conditions does embryonic growth occur post-dispersal? We predicted that germination of *A. glacialis* would decrease with decreasing cold stratification duration, as reported for *Aegopodium podagraria* (Apiaceae) seeds from Europe (Vandelook *et al.*, 2009). We also predicted that *A. glacialis* seeds from populations at lower elevations would be adapted to shorter durations of winter and therefore respond better to shorter durations of cold stratification for dormancy alleviation than seeds from higher elevations. Such a pattern was reported for *Phacelia secunda* (Boraginaceae) seeds collected in the Chilean Andes (Cavieres and Arroyo, 2000). We were also interested to know whether there would be any cost to seedlings that did germinate after a shortened winter, e.g. in terms of reduce growth rate, compared with seedlings that received a ‘normal’ duration of winter temperatures. Based on knowledge of other Apiaceae species, we predicted that *A. glacialis* seeds would possess underdeveloped embryos at dispersal (i.e. exhibit morphophysiological dormancy).

## Materials and methods

### Seed collection, moisture content and viability

*Aciphylla glacialis* (Apiaceae) grows in clumped populations in herbfields across much of the Australian alpine zone (Powell, 2011). Sites are characterized by winter snow cover, generally beginning in June and extending through October or November, during which time air temperatures are commonly below zero. Temperatures average between 15 and 20°C during summer, and higher elevation sites have colder mean temperatures and also less frequent extreme heat and cold events (Briceño *et al.*, 2014). Average soil temperature does not drop below freezing under snow during winter where stable vegetation cover exists (G. L. Hoyle *et al.*, unpublished observations).

Seeds were collected in March 2011 from six different *A. glacialis* populations, at altitudes ranging from 1927 to 2197 m above sea level, representing the range of elevations at which *A. glacialis* occurs in Kosciuszko National Park, New South Wales, Australia (Table 1). Low- and high- elevation sites were selected as pairs along three transects, namely Mt Kosciuszko, Mt Stilwell and Mt Albina. Populations consisted of between 30 and 200 fruiting individuals distributed across an area ∼200 m^2^. Seeds were collected by hand from at least 30 adult plants per population and were brown in colour and at the point of natural dispersal. Post-collection, seeds were bulked per population and stored in the laboratory (∼20°C, 45% relative humidity) for up to 5 days before being moved into experiments or stored in a drying room (see below).

**Table 1:** *Aciphylla glacialis* population details, seed moisture content and tetrazolium chloride-estimated viability prior to germination experiments

Population	Collection date	Elevation (m above sea level)	Aspect	No. of plants sampled	Seed moisture content (% ± SE, *n* = 3)	Seed viability (% ± SE, *n* = 3)
Mt Stilwell-low	29 March 2011	1927	W, steep	42	64 (±4)	100 (±0)
Merritt's creek	2 March 2011	1944	Flat, open	202	36 (±2)	94 (±3)
Mt Stilwell-high	29 March 2011	2037	SE, steep	50	70 (±1.5)	97 (±3)
Lake Albina	2 March 2011	2046	N, steep	120 +	34 (±1)	97 (±3)
Mt Kosciuszko-low	2 March 2011	2058	SE, steep	30 +	34 (±1)	88 (±4)
Mt Kosciuszko-high	15 March 2011	2197	W, steep	89	49 (±5)	94 (±3)

Seed moisture content was assessed for each population within 3–5 days of collection. Three replicates of 10 full and undamaged seeds per population were weighed, dried at 100°C for 18 h in a laboratory oven (Thermoline Scientific, Melbourne, NSW, Australia), and then re-weighed. Seed moisture content [(initial weight minus dry weight)/initial weight] ranged from 34 ± 1 to 70 ± 1.5% across the six populations (mean ± SEM; Table 1). Seed viability was assessed for each population 1–4 weeks post-collection (seeds were stored in a dry room at 15°C and 15% relative humidity until tests began), using the tetrazolium chloride staining technique (International Seed Testing Association, 2003). Three replicates of 36 randomly selected seeds per population were hydrated in Petri dishes containing 1% plain water agar for 24 h at room temperature (∼20°C), before being scarified, away from the embryonic axis, and placed in 1% tetrazolium chloride solution in a dark, 30°C oven for 24 h. Seeds were then cut in half and embryos examined. Only uniformly stained red/dark pink embryos were considered ‘viable’ (Hoyle and PrometheusWiki contributors, 2012). Seed viability ranged from 88 ± 4 to 100 ± 0% per population (mean ± SEM; Table 1).

### Effect of cold duration on germination and early seedling growth

To investigate the effect of reduced lengths of cold duration on germination percentage, four replicates of 18 seeds (including the surrounding pericarp), per population, per cold duration, were sown into plastic Petri dishes 9 cm in diameter containing 1% plain water agar. Petri dishes were sealed using Parafilm before being placed in the germination incubators (Thermoline Scientific, Wetherill Park, NSW, Australia). Each replicate was placed on a different incubator shelf and re-randomized on that shelf weekly. A constant 5.1 ± 1.8°C (mean ± SD) temperature regimen was selected to approximate temperatures that dispersed seeds would experience *in situ* during winter under snow (Briceño *et al.*, 2014). Six cold-temperature treatments of decreasing duration (10–0 weeks of incubation at 5°C in 2 week increments) were established. During cold treatment, Petri dishes were wrapped in aluminium foil to simulate dark beneath snow and compacted herbfield (Richardson and Salisbury, 1977). Following the cold period, aluminium foil was removed and the Petri dishes were moved to a warmer temperature regimen, with day–night temperatures designed to approximate temperatures that seeds would experience *in situ* during early spring, i.e. 9.9 ± 1.7°C during the day and 5.2 ± 2.1°C at night (mean ± SD). In this temperature regimen a 12 h–12 h light–dark photoperiod was provided by fluorescent tubes (∼50 µmol m^−2^ s^−1^).

Each week, seeds were checked for germination, defined as >1 mm of visible radicle. Where light was excluded from seeds, germination was assessed in a dark room under a dim green light. Seedlings were removed from the dishes following germination and discarded or grown on (see growth experiment below). The experiment was terminated at 14 weeks when no further germination from any population had occurred for at least 2 weeks. Any remaining intact seeds were dissected with a scalpel under a microscope, and those containing a firm, fresh endosperm and embryo were deemed to be still viable. Seeds empty of an embryo or infected by fungus were deducted from the total when calculating the percentage germination for each population.

The first four *A. glacialis* seeds to germinate per Petri dish were grown on in order to monitor early seedling development. Seedlings were placed on a surface of composted coir potting mix (trace elements added; Australian National Botanic Gardens mix) and supplied with two pellets of Yates Nutricote Grey fertilizer (16N 4.4P 8.3K; Yates Australia, Padstow, NSW, Australia) in plastic pots 40 mm wide. Seedlings were then grown in a glasshouse at 10/6°C day/night temperatures with natural light (day length effectively the same as in nearby alpine area) and watered approximately three times each week, or as needed to keep the soil moist. Seedlings were randomly arranged in trays that were re-organized on the glasshouse bench weekly; pots were re-arranged periodically within trays. Seedling emergence (defined as the time it took for at least one cotyledon to become separated from the pericarp) and leaf emergence (defined as the point where the first junction between leaf pinnae was visible) were recorded weekly for 8 weeks, at which point plant height was also measured.

### Effect of temperature and light on embryonic growth

To investigate whether *A. glacialis* has morphological dormancy, embryonic growth inside seeds of two populations (Mt Stilwell, high and low) was assessed in incubators (see previous subsection) using a factorial design consisting of two temperature treatments (a constant 5°C in darkness, mimicking winter; and alternating 10–5°C, 12 h–12 h with 12 h photoperiod, mimicking late summer or early spring conditions, ∼50 µmol m^−2^ s^−1^). Three replicates of 30 seeds per population, per treatment, were sown into plastic Petri dishes 9 cm in diameter containing 1% plain water agar. Petri dishes were sealed using Parafilm and placed in treatment conditions. Five seeds were removed from each dish 1–2 days after the start of the experiment, and every 2 weeks thereafter, and were dissected under a microscope (Lynx Dynascope; Vision Engineering Ltd, Woking, Surrey, UK). One population was assessed each week in alternate weeks. For each seed, the pericarp was removed and an image of the endosperm and the embryo, both removed from the seed, was captured using Amcap and still capture, version 9.20 (Amcap Directshow Video; Afterdawn Oy, Oulu, Finland). The length of each embryo and endosperm was then measured, using ImageJ software (Rasband, 1997–2014). Any germinated seeds, and those disfigured in the dissection process or discovered to be mould infested, were excluded.

### Data analysis

All analyses were performed using GenStat 14th edition (VSN International, Hemel Hempstead, Hertfordshire, UK). The final germination percentage was calculated as the percentage of full and viable seeds germinating per dish. The final germination percentage was compared between populations, cold duration and replicate shelves using restricted maximum likelihood linear regression analysis. The Mt Stilwell-high population had very low germination (<5%) across all cold durations, as did seeds of all populations that received no cold stratification (0 weeks); these treatments were excluded from the statistical analysis. Initial models included elevation and duration as fixed factors, as well as transect and shelf within the incubator as random factors. Elevation and transect both proved to be non-significant factors in determining the final germination percentage and, when included as a factor in the regression analysis, did not accurately model the data for all populations. Elevation and transect were therefore replaced by the fixed term, population, in the final model. Incubator shelf was included in the random as well as the fixed model and was significant in all tests.

Seedling growth traits were analysed using a generalized linear regression model for unbalanced designs. Factors in the model were incubator shelf (random), population, cold duration and the interaction between population and cold duration.

For the morphological dormancy experiment, results for alternate weeks were bulked (e.g. 1 and 2 weeks) in order to include data from both populations. Embryonic length was log transformed to meet assumptions of normality. Embryonic growth in seeds from the two populations and four experimental conditions was compared using restricted maximum likelihood linear regression analysis, where endosperm length was used as a covariate to account for differences in initial seed size. We first confirmed that endosperm size itself was not significantly affected by time, light or temperature (results not shown). Terms in the model included endosperm length, shelf, population, temperature, light and time.

## Results

### Effect of cold duration on seed germination and early seedling growth

#### Seed germination

**Figure 1:**
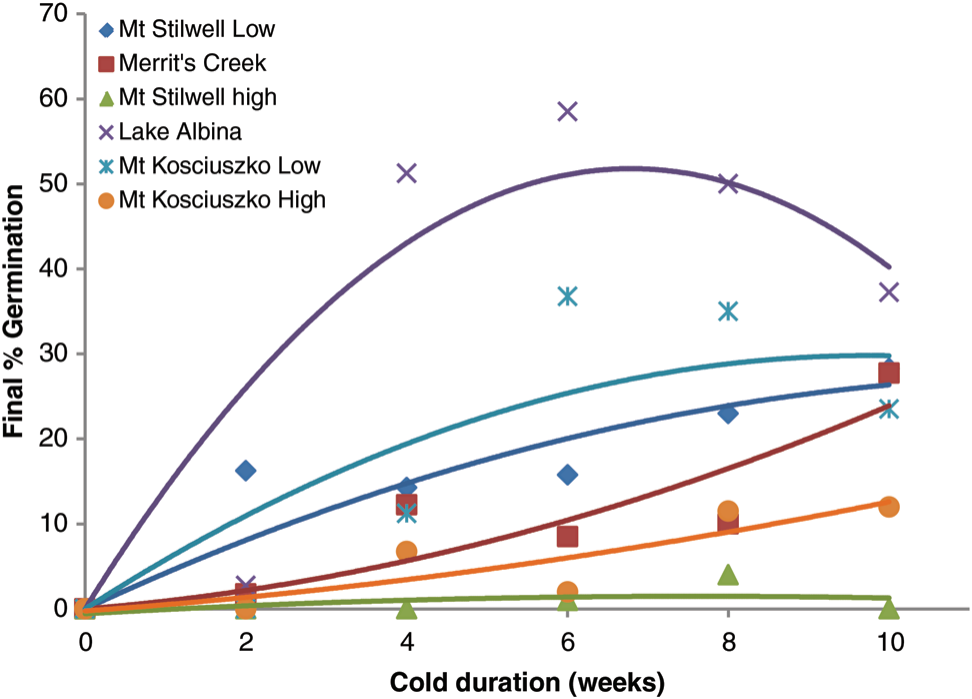
Effect of cold duration (constant 5°C, dark) on mean final seed germination in six populations of *Aciphylla glacialis.* There was no germination of seeds that did not receive any cold stratification. The polynomial line was fitted to aid visual comparison only. Sites of seed origin are listed from lowest (1927 m above sea level) to highest elevation (2197 m above sea level); see Table 1.

**Figure 2:**
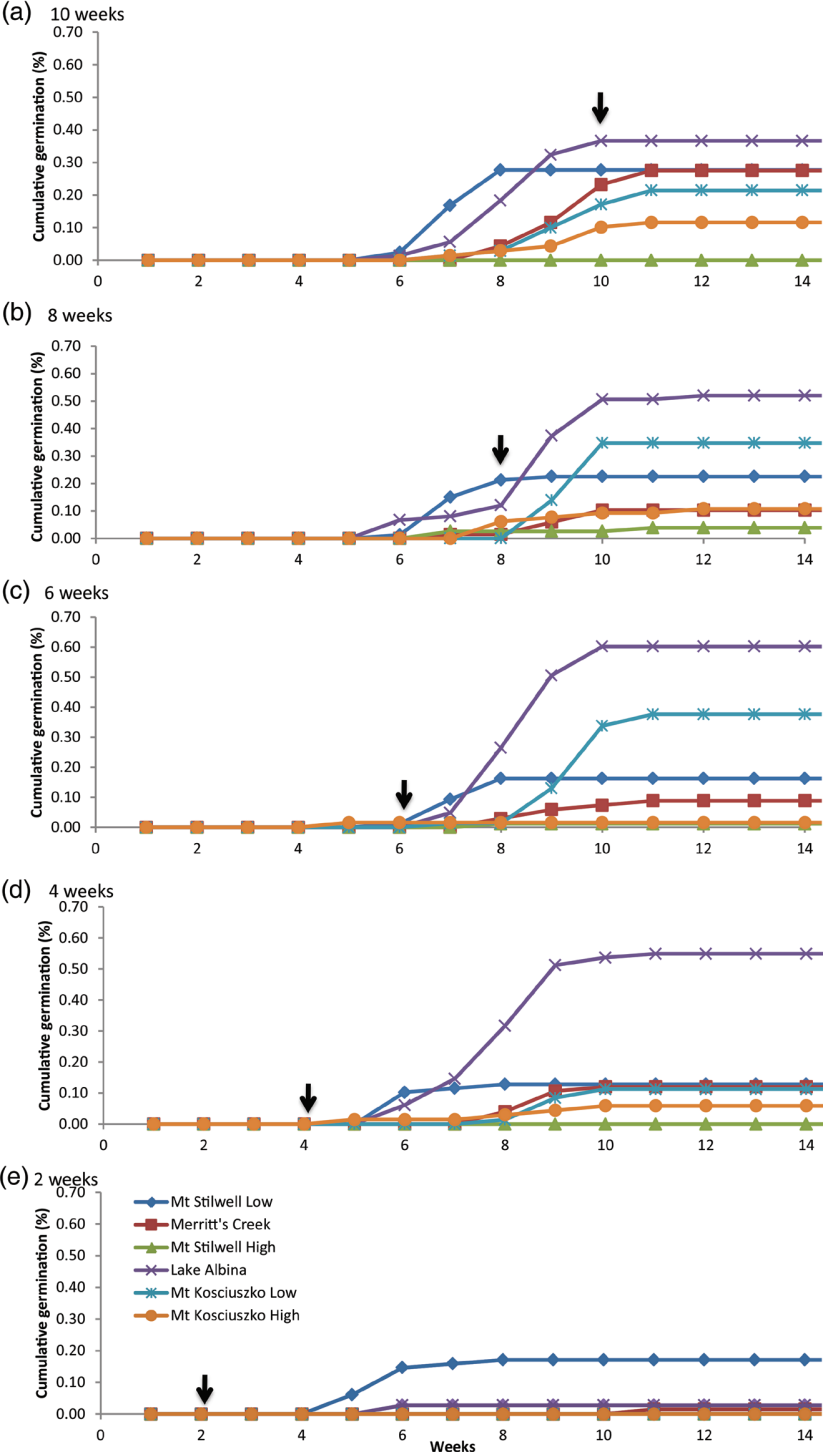
Effect of progressively shorter cold duration (constant 5°C, dark) on cumulative percentage germination of *A. glacialis* seeds collected from six populations. Arrow indicates when seeds were moved to 10–5°C, 12 h–12 h light–dark photoperiod. Sites of seed origin are listed from lowest (1927 m above sea level) to highest elevation (2197 m above sea level); see Table 1 for details.

*Aciphylla glacialis* seeds required cold conditions (5°C) for germination; seeds placed directly at 10–5°C did not germinate, regardless of population (data not shown). Contrary to expectations, a small number of seeds from three populations germinated when moved to 10–5°C following only 2 weeks at 5°C, in particular, seeds from the lowest elevation site (Mt Stilwell-low; Figs 1 and 2a). Seeds from Lake Albina and Mt Stilwell-low germinated earlier than seeds from other sites, regardless of cold duration (Fig. 2). The final percentage germination increased with cold duration for four of the six populations (Fig. 2); for the remaining two populations (Lake Albina and Mt Kosciuszko-low), germination was lower following cold duration exceeding 6 weeks (Fig. 1). Note that while both population and duration effects were statistically significant (*P* < 0.000 and <0.008, respectively; Supplementary Table 1a), the population-by-duration interaction was not. In addition, contrary to our initial prediction, populations with the highest and lowest final percentage germination were both of intermediate elevation (Fig. 2). We noted that when high- and low- elevation seeds of Mt Kosciuszko and Mt Stilwell were compared, however, seeds from the higher sites appeared somewhat more dormant (i.e. achieved lower germination across experimental conditions) than seeds from the lower-elevation sites on the same mountain. Regardless of population and temperature, virtually no further germination occurred after 10 weeks of incubation (Fig. 2). Notably, the maximal percentage germination for all populations was relatively low (<60%), despite high tetrazolium chloride-estimated viability (>88%).

#### Early seedling growth

Seedlings from the germination experiment were grown in common glasshouse conditions to assess whether cold duration affected early seedling growth. While populations differed significantly in seedling growth and development rates, there was no evidence that seeds germinating following shorter cold periods were disadvantaged in seedling growth traits; there were no significant cold duration effects, nor were the interactions with population or cold duration significant (Supplementary Table 1b).

### Effect of temperature and light on embryonic growth

**Figure 3:**
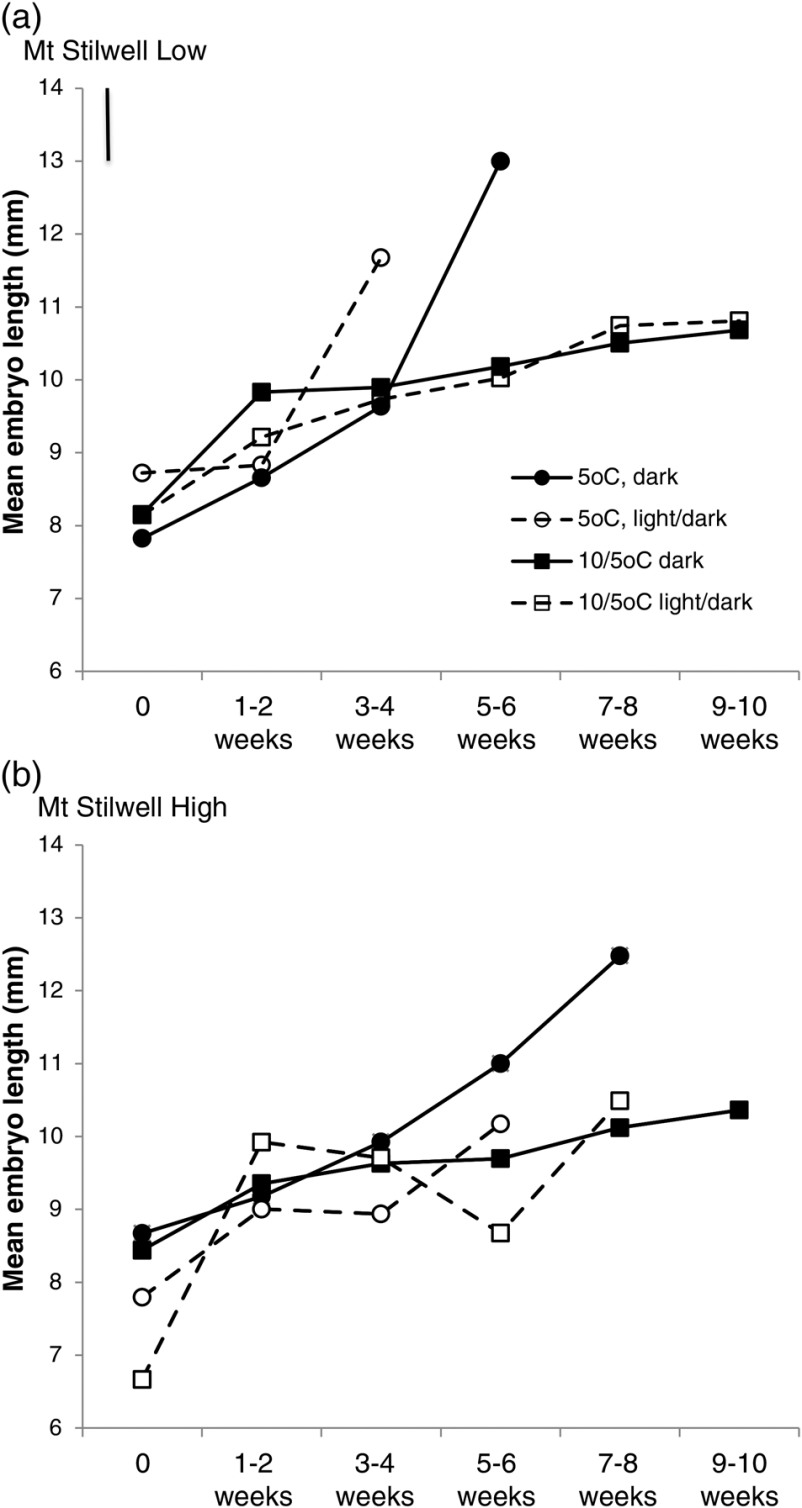
Effect of temperature and light on *A. glacialis* embryonic growth inside seeds from Mt Stilwell-low (1927 m above sea level; **a**) and Mt Stilwell-high populations (2037 m above sea level; **b**), prior to germination. Data are plotted until germination begins, at which point sample size declines and the length of embryos inside remaining, ungerminated seeds was deemed not necessarily representative. Verticle bar in panel (a) denotes least significant difference.

Seeds of *A. glacialis* showed evidence of underdeveloped embryos at dispersal, and the growth rate of the embryos varied with temperature and light conditions (Table 2). There were no significant population effects or interactions (Table 2); seeds had an initial embryo-to-endosperm ratio of 0.15 (±0.07) and a final ratio of 0.21 (±0.1) prior to germination, averaged across populations and treatments. Embryos began to grow immediately in all treatments, increasing significantly in length over time at both constant 5°C and alternating 10–5°C, in both constant dark and alternating light–dark (Fig. 3). Seeds at 5°C went on to germinate within 4–5 weeks (Mt Stilwell-low) or 8–9 weeks (Mt Stilwell-high; Fig 3).

**Table 2:** Effects of cold duration and light on embryonic length in *A. glacialis* seeds as determined by restricted maximum likelihood linear regression analysis

Parameter	Wald statistic	n.d.f.	*F* statistic	d.d.f.	*F* pr
Endosperm	17.25	1	17.25	575	<0.001*
Shelf	3.2	2	1.6	27.3	0.221
Population	4.03	1	4.03	27.6	0.054
Temperature	6.56	1	6.56	560	0.011*
Light	4.03	1	4.03	559.2	0.045*
Time	85.18	5	17.03	27.7	<0.001*
Population × light	3.57	1	3.57	557	0.06
Temp × time	18.84	5	3.77	559.9	0.002*

Endosperm length was used as a covariate. All interactions were included in initial model and non-significant interaction terms removed starting from highest-order interactions. *Significant (*P* < 0.05).

Abbreviations: d.d.f., denominator degrees of freedom; n.d.f., number of degrees of freedom.

## Discussion

Our results support previous findings that seeds of the Australian alpine herb *A. glacialis* (Apiaceae) possess dormancy at the time of dispersal. In addition to physiological dormancy that was alleviated by cold stratification, we found evidence of underdeveloped embryos at dispersal, consistent with the prediction of morphological dormancy. Embryos grew at both constant 5°C and alternating 10–5°C but did not germinate at the higher temperature, indicating that the morphological component of dormancy can be alleviated at a range of temperatures but cold stratification is required to alleviate the physiological component of dormancy and, ultimately, enable germination. Across populations, seed dormancy status differed significantly but, contrary to predictions, elevation did not explain the variation. Seeds began germinating after 4–6 weeks at 5°C, and seedling vigour did not depend on the duration of the cold period. Our results suggest that projected changes in winter duration are unlikely to disrupt germination or early seedling growth directly in this species. Here, we discuss the implications of a germination strategy involving morphophysiological dormancy, and the demonstrated differences among populations, in the context of predicting the impact of climate change upon the future germination success of *A. glacialis* and other alpine species.

### Seed germination strategy

Germination of *A. glacialis* at 10–5°C without prior cold treatment was prevented by physiological dormancy. The 10–5°C temperature regimen was selected to approximate *in situ* temperatures experienced by *A. glacialis* soon after seeds are dispersed (autumn) and soon after snowmelt following winter (spring). Results suggest that *A. glacialis* seeds, although dispersed in summer and early autumn, postpone germination until during or following the subsequent winter. Germination occurred both at 10–5°C following cold stratification and at temperatures reflecting those below the snow during winter (5°C), indicating that cold itself does not inhibit germination (G. L. Hoyle *et al.*, unpublished observations). Rather, the overall requirements for dormancy alleviation and germination suggest an adaptation to trigger germination in late winter (Scholten *et al.*, 2009). We also conclude that *A. glacialis* does not have a light requirement for germination; the alleviation of both morphological dormancy and physiological dormancy, and the commencement of germination, occurred in both alternating light–dark and constant darkness, supporting the idea that seedlings may emerge under snow cover. In contrast, other Apiaceae species require light to promote embryonic growth (Baskin and Baskin, 1990; Baskin *et al.*, 2004; Vandelook *et al.*, 2008). Postponing germination until seeds are under snow cover would enable seedlings to optimize the short forthcoming growing season and may also decrease the risk of frost damage during the early seedling stage (Drescher and Thomas, 2013). Vandelook *et al.* (2012) concluded that evolution towards germination at low temperatures has occurred in Apiaceae species growing in cold regions; however, whether *A. glacialis* seedlings do survive beneath the snow *in situ* has yet to be confirmed.

Embryonic growth to the critical size for germination required at least 4 weeks, in contrast to other morphologically dormant species that require between 8 and 22 weeks (Vandelook *et al.*, 2009). This relatively fast embryonic growth prior to germination suggests that *A. glacialis* seeds possess large embryos at dispersal, and may also explain why the final embryo-to-endosperm ratio is relatively low in *A. glaciallis* compared with some other species (e.g. Vandelook *et al.*, 2007, 2009). Alternatively, embryonic growth may have accelerated in the final days to germination, so that we underestimated it with our weekly sampling. Nonetheless, the results show that the growth was significant and necessary for germination.

Large embryonic size is considered beneficial in dry habitats, where rapid germination during short wet periods is advantageous (Hodgson and Mackey, 1986), but may also benefit seeds in short and unpredictable alpine growing seasons. Winter temperatures alleviated both physiological dormancy and morphological dormancy of *A. glacialis* simultaneously. Although embryonic growth occurred at both 5°C and 10–5°C, the results suggest that embryonic growth at 10–5°C was not sufficient for germination because physiological dormancy had not been alleviated. Cold temperatures have been shown to alleviate morphological and physiological dormancy simultaneously in other Apiaceae species, such as *Lomatium dissectum* (Scholten *et al.*, 2009) and *Ozmorhiza aristata* (Walck *et al.*, 2002). In contrast, in two widespread European Apiaceae species, *Angelica sylvestris* and *Selinum carvifolia*, embryonic elongation happens at warm temperatures (20–23°C) but only after physiological dormancy has been alleviated by cold stratification (Vandelook *et al.*, 2007). Mechanisms that support embryonic growth at low temperatures may include the availability of nutrient reserves, the mobilization of sugars and/or a role for the hormone gibberellic acid in endosperm breakdown (Vandelook *et al.*, 2009). *In situ*, dispersal of underdeveloped embryos would ensure that *A. glacialis* germination is postponed even in the event of a ‘cold snap’ that could alleviate physiological dormancy soon after seed dispersal. In addition, dispersal of morphologically dormant seeds may be a mechanism for reducing seed loss to granivores while on the plant.

For two of the six populations, spending longer than 6 weeks at 5°C appeared to reduce the final percentage germination and, despite high viability, virtually no germination of any population occurred after 10 weeks of incubation. These results suggest that seeds had cycled back into physiological dormancy. Dormancy cycling in the soil seed bank is common and can contribute to the persistence of seeds through time (Fenner and Thompson, 2005; Mennan and Zandstra, 2006), particularly in the absence of a light requirement for germination. The ability to cycle in and out of dormancy would add another level of conservatism to the *A. glacialis* germination strategy.

### Effect of predicted climate change on the regeneration success of ***A. glacialis***

Winter in the Australian Alps is traditionally characterized by snow cover beginning in June; however, since records began in 1954, spring thaw has been occurring, on average, 2 days earlier per decade (Green and Pickering, 2009). It is therefore worth reflecting on how the germination strategy of *A. glacialis* might influence its performance under shortened winters. We have shown that dormancy alleviation of *A. glacialis* can occur in a relatively short time; surprisingly short durations of cold temperatures were enough to elicit germination of some seeds. In contrast, at least 8 weeks of cold stratification were required to elicit germination of the morphophysiologically dormant species *A. podagraria* seeds, and even after 12 weeks, germination was still low (6%; Vandelook *et al.*, 2009). In addition, our results suggest that early seedling development is likely to remain unaffected by changes in cold temperature duration prior to germination. We found no evidence that reduced periods for dormancy alleviation impacted upon early seedling growth, as it might if abscisic acid-to-gibberellic acid ratios were altered with shorter periods of cold. In conclusion, shorter winters *per se* are unlikely to disrupt the germination strategy of this alpine herb. A shift from spring to autumn germination due to autumn warming (Mondoni *et al.*, 2012) is unlikely for *A. glacialis* given the morphological dormancy. Despite this, cold temperatures are crucial to dormancy alleviation of *A. glacialis* seeds; therefore, without further investigation, particularly *in situ*, we cannot rule out the possibility that predicted warmer alpine temperatures (Hennessy *et al.*, 2003) could compromise dormancy alleviation of this species.

Populations differed in their response to cold stratification, both in final germination and in embryonic development, suggesting that dormancy status, and thus germination strategy, differed between populations. We hypothesized that seeds from higher elevation sites might be more dormant, i.e. require longer periods of cold stratification for germination, than seeds from lower elevations, but we found no overall correlation between elevation and germination. There are many other potential causes of variation among populations, such as soil conditions, aspect, population age and small-scale genetic differentiation among populations (due to either local adaption or drift). Seasonal variation in environmental conditions may also lead to significant differences in maternal provisioning of seeds, and this could have a significant effect on dormancy status and/or germination requirements at dispersal (Donohue, 2005; Fenner and Thompson, 2005; Hoyle *et al.*, 2008a). In addition, we observed that seed moisture content at dispersal varied among populations, suggesting that although all seeds were at the point of natural dispersal when collected, they may have varied in maturity, which may also contribute to differences in dormancy status between populations (Hay and Smith, 2004). Lastly, the low germination relative to viability may be explained by light quality/availability or by a need for warm stratification prior to cold stratification (Vandelook *et al.*, 2008, 2009). These caveats aside, the marked population variation that was observed suggests that dormancy status, and thus the timing of germination, are highly plastic traits within *A. glacialis*.

### Implications of warming climate and dormancy strategies for other alpine plant species

Recent work demonstrates that dormancy is common in the Australian alpine flora (G. L. Hoyle *et al.*, unpublished observations). The prevalence of morphological dormancy or morphophysiological dormancy in the Australian flora is little known, although morphological dormancy is common in the Apiaceae, Ranunculaceae and Liliaceae families (Baskin and Baskin, 2001), of which there are several species of ecological and cultural significance in the flora. For a large number of Australian alpine species, a single-population seed collection may contain both dormant and non-dormant seeds (G. L.Hoyle *et al.*, unpublished observations). The causes of this variation, like the variation among populations shown for *A. glacialis* here, remain poorly understood but could include differences in flowering time, seed mass, parental investment, climate, elevation and/or maternal environment driven by genetic, epigenetic and/or environmental factors (Cochrane *et al.*, in press). For species with morphological dormancy, the variation may also reflect an interaction between processes of morphological and physiological dormancy alleviation. Further research into how changes in temperatures, reduced duration of snow cover and increased frequency of extreme temperature events will affect species with varied dormancy syndromes are required in order to predict how species with morphological and/or physiological dormancy, in particular, will respond to climate change.

Identification of within-species variation in seed germination and dormancy traits may assist with conservation, management and plant community restoration actions that buffer against loss of diversity (Broadhurst *et al.*, 2008) and support more precise forecasting of the vulnerability of species to climate change (Cochrane *et al.*, in press). In particular, determining the cause and distribution of variation in germination strategies will assist in selecting material for restoration programmes. Therefore, the generality of the variation observed in *A. glacialis* deserves further attention, as does the mechanism by which variation in dormancy status is invoked across, as well as within, species.

## Supplementary material

Supplementary material is available at *Conservation Physiology* online.

Supplementary Data
