# Supplementary material for: Effects of reduced winter duration on seed dormancy and germination in six populations of the alpine herb Aciphyllya glacialis (Apiaceae)
Source: Conserv Physiol. 2014 May 30;2(1):cou015. doi: 10.1093/conphys/cou015 (PMC4806741; doi:10.1093/conphys/cou015)
Supplement: Supplementary Data [file supp_2_1_cou015__index.html]

Effects of reduced winter duration on seed dormancy and germination in six populations of the alpine herb Aciphyllya glacialis (Apiaceae) — Supplementary Data 

# Effects of reduced winter duration on seed dormancy and germination in six populations of the alpine herb *Aciphyllya glacialis* (Apiaceae)

## Supplementary Data

Supplementary Data

**Files in this Data Supplement:**

- Supplementary Data - Docx file
