# Supplementary material for: Effects of reduced winter duration on seed dormancy and germination in six populations of the alpine herb Aciphyllya glacialis (Apiaceae)
Source: Conserv Physiol. 2014 May 30;2(1):cou015. doi: 10.1093/conphys/cou015 (PMC4806741; doi:10.1093/conphys/cou015)
Supplement: Supplementary Data [file supp_cou015_cou015supp.docx]

# Supplementary table 1.

a) Results of Residual Maximum Likelihood analysis on final percentage germination of *Aciphylla glacialis* seeds.

| Factor | Wald statistic | n.d.f. | F statistic | Final % germination |
| --- | --- | --- | --- | --- |
| Shelf | 9.43 | 3 | 3.14 | **0.030** |
| Population | 28.44 | 4 | 7.11 | **<0.001** |
| Cold duration | 14.87 | 4 | 3.72 | **0.008** |
| Population × cold duration | 16.60 | 16 | 1.04 | 0.430 |

b) General Linear Model results showing factors affecting seedling traits in five populations of *A. glacialis* seeds.

| Parameter | df | Days to cotyledon emergence | Time to first leaf | Height at week 8 |
| --- | --- | --- | --- | --- |
| Shelf | 3 | 0.774 | 0.353 | **0.021** |
| Population | 4 | **0.023** | **0.009** | **0.010** |
| Cold duration | 3 | 0.233 | 0.461 | 0.988 |
| Population × Cold duration | 12 | 0.532 | 0.848 | 0.436 |

# Supplemental figure S1


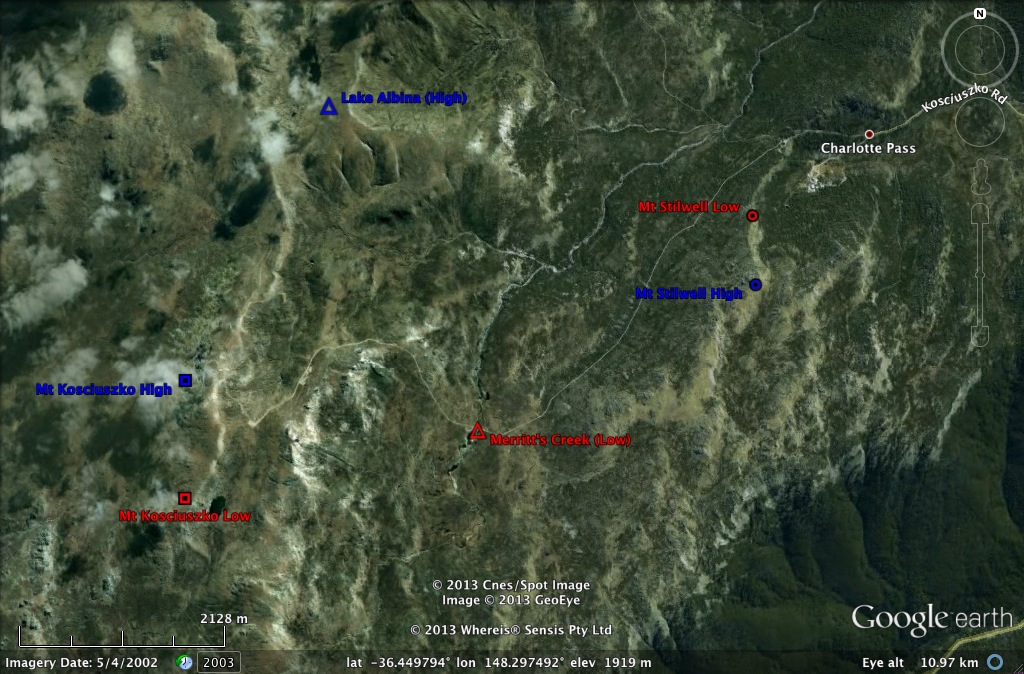

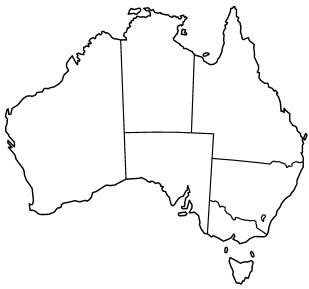


**Figure S1** Map of population locations, Kosciuszko National Park, NSW. The village of Charlotte Pass is included as a reference point. Blue points are the high elevation and red points the low elevation end of each of three paired transects: Mt Kosciouszko (squares), Lake Albina/Merritt’s Creek (triangles), and Mt. Stillwell (circles). Blue star on inset map marks study site.
